# A new, short-recorded photoplethysmogram dataset for blood pressure monitoring in China

**DOI:** 10.1038/sdata.2018.20

**Published:** 2018-02-27

**Authors:** Yongbo Liang, Zhencheng Chen, Guiyong Liu, Mohamed Elgendi

**Affiliations:** 1 School of Electronic Engineering and Automation, Guilin University of Electronic Technology, Guilin 541004, PR China; 2 School of Life and Environmental Sciences, Guilin University of Electronic Technology, Guilin 541004, PR China; 3 School of Electrical and Computer Engineering, University of British Columbia, Columbia, Vancouver V6T 1Z4, Canada; 4 Guilin People's Hospital, Guilin 541000, PR China; 5 Department of Obstetrics & Gynecology, University of British Columbia, Columbia, Vancouver V6H 3N1, Canada; 6 BC Children's & Women's Hospital, Vancouver, Vancouver V6H 3N1, Canada

**Keywords:** Diagnostic markers, Predictive markers, Hypertension, Diagnostic markers

## Abstract

Open clinical trial data provide a valuable opportunity for researchers worldwide to assess new hypotheses, validate published results, and collaborate for scientific advances in medical research. Here, we present a health dataset for the non-invasive detection of cardiovascular disease (CVD), containing 657 data segments from 219 subjects. The dataset covers an age range of 20–89 years and records of diseases including hypertension and diabetes. Data acquisition was carried out under the control of standard experimental conditions and specifications. This dataset can be used to carry out the study of photoplethysmograph (PPG) signal quality evaluation and to explore the intrinsic relationship between the PPG waveform and cardiovascular disease to discover and evaluate latent characteristic information contained in PPG signals. These data can also be used to study early and noninvasive screening of common CVD such as hypertension and other related CVD diseases such as diabetes.

## Background and Summary

The incidence of cardiovascular disease (CVD) has risen around the world in recent years overtaking the mortality rate of cancer, making CVD the number one killer of humans. Many studies have been conducted using noninvasive early diagnosis and screening techniques for CVDs such as hypertension and coronary artery sclerosis in order to discover more convenient and effective methods for the early identification of CVDs. Of these methods, the photoplethysmography (PPG) has become widely recognized as a low-cost non-invasive detection technology for CVDs. The cardiovascular parameters detected using PPG technology include heart rate, blood oxygen saturation, blood pressure, assessment of arterial stiffness^1^, and pulse wave velocity, among others^2^. The PPG signal includes information on the hemodynamic process^3,4^, hemorheology^5^, and tissue status of the peripheral microcirculation system in the human body^6^. That is, the PPG signal is an aggregated expression of many physiological processes in the cardiovascular circulation system^7^. A physiological information database with high precision and a high sampling rate is urgently needed in PPG technology research in order to extract more cardiovascular parameters for the early screening and diagnosis of CVDs. We provide here a database containing physiological information and PPG waveform data collected over a year that can be used to research arterial blood vessel aging, arterial blood pressure detection^8^, and screening of hypertensive and diabetic patients based on PPG signals.

Electrocardiogram (ECG) signal and PPG signal can effectively estimate blood pressure which has been recently studied improved by researchers^9^. However, there are some limitations with collecting ECG and PPG simultaneously using a mobile phone^10^. For these reasons, and for simplicity, a few researchers have attempted to estimate blood pressure based using only PPG signals^11^. The concept of estimating blood pressure (BP) using only PPG signals seems to promising and is optimally implemented when the PPG signal is of high quality. Therefore providing a database that can help with estimating BP using only PPG will help further research in this area.

This PPG and BP (PPG-BP) database integrates the deidentified, comprehensive clinical data of patients admitted to the Guilin People's Hospital in Guilin, China. The openness of the data allows clinical studies to explore and improve the understanding of relationships between cardiovascular health and PPG signals, with the final goal of creating a simple, effective non-invasive detection technology that is easy to use and wearable. This dataset has been collected from 219 subjects, aged 21–86 years, with a median age of 58 years. Males accounted for 48%. The dataset covers several diseases including hypertension, diabetes, cerebral infarction, and insufficient brain blood supply.

In summary, this unique non-invasive detection dataset for cardiovascular disease can be used in a wide range of in-depth research. In the following section, we describe the database detail records, database usage descriptions, and we also explain how to fully use the dataset. We also provide an example of the basic properties of the database that allows researchers to conduct research.

## Methods

### Experimental design and data acquisition

The dataset collection program involved acquiring information on the basic physiology of individuals, extracting information on cardiovascular diseases from hospital electronic medical records, collecting PPG waveform signals, and detecting instant arterial blood pressure at the same time. The data acquisition was conducted at the Guilin People's Hospital.

A customized portable hardware platform was designed, and consisted of a PPG senor probe, microcontroller, and a matching app. Data were transmitted via Bluetooth. The PPG sensor model was SEP9AF-2 (SMPLUS Company, Korea), which contains dual LED with 660nm (Red light) and 905 nm (Infrared) wavelengths, with a sampling rate of 1 kHz and 12-bit ADC, and the hardware filter design is 0.5‒12Hz bandpass. The microcontroller model was MSP430FG4618 (Texas Instruments company, USA) embedded on the probe’s board to configure the ADC, fetch the data and send the data to the matching app via Bluetooth.

Waveform data is collected using a set of customized probes and a matching app that was developed based on Android Studio. The PPG detection probe used the infrared light and transmission method to collect fingertip PPG waveform data. These real-time data are transmitted to the matching app via Bluetooth. The app can control the detection probe, display the real-time waveform, and conduct a signal quality assessment of the PPG waveform in order to save the high-quality PPG wave segment. The arterial blood pressure is measured using the Omron HEM-7201 (Omron Company, Kyoto, Japan) upper arm blood pressure monitor, which is validated in ref. ^12^.

The study was approved by the ethics committee of the Guilin People Hospital and the Guilin University of Electronic Technology in China. All participants gave written and informed consent before the study. They were compensated monetarily at 10 Yuan/h. Participants answered questions about age, gender, height, and weight and all initial data acquisition was conducted in a private, and comfortable clinical room.

**Figure 1:**
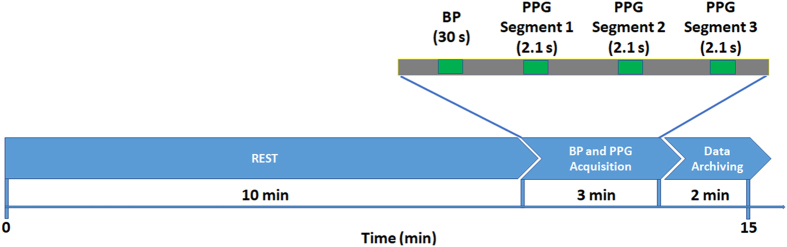
Measurement Protocol. The duration of the whole experiment was approximately 15 minutes. The photoplethysmogram (PPG) and blood pressure (BP) were collected within 3 minutes. Three PPG segments collected per subject, with a duration of 2.1 seconds each.

As shown in Figure 1, before beginning with signal collected, each individual was asked to sit in an office chair in the most comfortable posture and to relax their arms on an empty desk. Each individual had 10 min to adapt to the environment and adjust their breathing after entering the data collection room. The specific collection settings were as follows: The PPG signal was collected at the fingertip of the left index finger, the arterial blood pressure was collected from the right forearm, all of which was completed within three minutes. The arterial blood pressure measurement was performed by the hospital nurse.

During signal acquisition the sampling precision of waveform data was set to a sampling rate of 1 kHz, with 12 bits AD conversion precision. Three segments were recorded and saved per subject, each segment included 2100 sampling points, which corresponds to a length of 2.1 seconds. During the 3 min data collection phase, every PPG segment of a particular subject scored a Skewness SQI value; values greater than Zero^13^ were saved, and if a value was less than Zero the app prompted the user to recollect the PPG signal. This step was developed to reduce including PPG segments with high noise and motion artifacts.

The BP collection device (Omron HEM-7201) requires at a minimum a 30 second waveform to detect the systolic and diastolic period. The BP reading represents the blood pressure value for the 3 minute data recording for each subject, as shown in Figure 1. During the data collection process, we aimed to collect the BP and PPG data immediately after each other respectively. Three PPG segments were saved during the data collection period in addition to the BP recording. Every participant was asked to breath as they normally would on day-to-day basis for practical applications. Note that we did not investigate the baroreflex response to stress. The dataset includes BP and PPG information from subjects that were diagnosed with normotension, prehypertension, and stage I/stage II hypertension, which can be helpful and valuable for researchers.

### Patient characteristics

**Figure 2:**
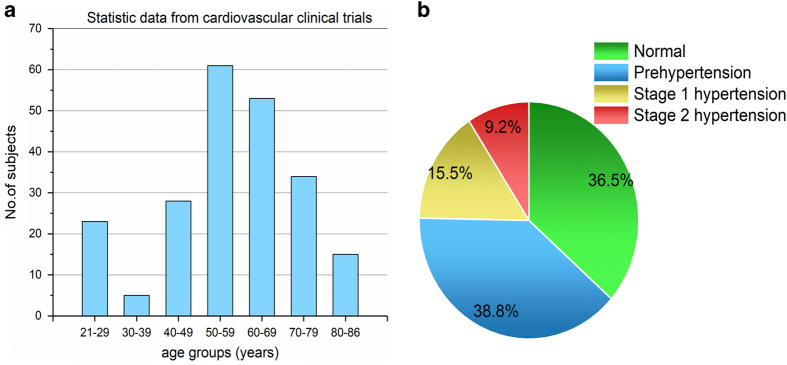
Statistics about the PPG-BP dataset. (**a**) histogram of age groups; (**b**) pie chart of blood pressure stages.

The dataset was collected from 219 adult subjects and currently contains 657 PPG waveform segments. The dataset covers individuals aged 21–86 years, and males account for 48% of participants. The dataset also covers several different CVDs, including hypertension, cerebral infarction, and insufficient brain blood supply and other related diseases such as diabetes. The statistical results are shown in Figure 2.

### De-identification

In the process of creating the dataset, the first task was to de-identify each participant and remove personal information such as name, telephone number, address, date, and so on.

### Data Records

The dataset has been fully uploaded to the network, and users can download them through the *Figshare* repository with the title *PPG-BP Database* and reference (Data Citation 1).

The dataset comprises 1 table file and 219 waveform data folders, which include three 2.1-second-length infrared PPG signal text files and physiological information recording files. Among these, the PPG signal data is the 2.1-second-length 12 bits AD raw value. The ID_1, ID_2, and ID_3 text files represent three separate segments of waveform data Table 1 (available online only).

The "PPG-BP database.xlsx" table file contains aggregated subjects of physiological information and disease information. Information records include ID, sex, age, height, weight, systolic pressure, diastolic pressure, heart rate, and disease records.

**Figure 3:**
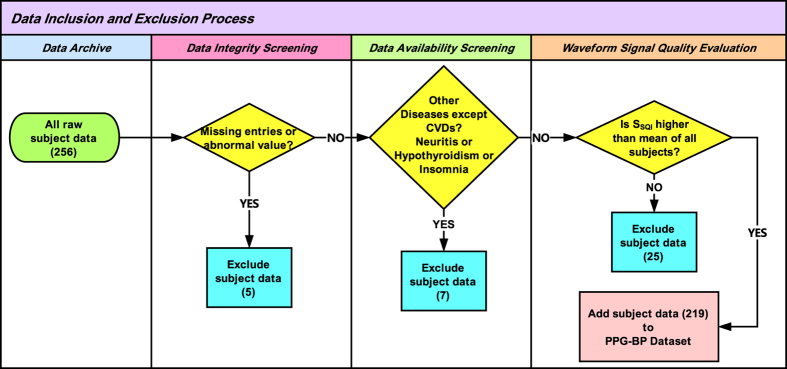
A process flowchart of data inclusion and exclusion. Note, S_SQI_ stands for Skewness signal quality index.

Before the participant record is archived, it was required to conduct data integrity screening, data availability screening, and a waveform signal quality evaluation (to remove inconsistent, abnormal, and high noise data) in order to form a high-quality dataset. The detailed process of inclusion and exclusion, as shown in Figure 3, is described as follows:

1. **Data integrity screening**: This process includes the screening of missing and abnormal values for: basic physiological information, disease information, blood pressure, heart rate, and 3 segments waveform data. If one or more items are missing or if there was an abnormal value, the participant record was removed.
2. **Data availability screening**: This dataset is designed to focus on the clinical information for CVDs and other closely related diseases such as diabetes. Data from the CVD patients who were diagnosed with non-CVD diseases (except diabetes) were excluded during the screening process to ensure that the dataset only contains data from participants who were diagnosed with the disease of interest.
3. **Waveform signal quality evaluation**: All 3 segments for each participant went through a signal quality evaluation, and a robust signal quality index (SQI) method was applied in order to achieve this step. If the SQIs of the 3 segments in one subject were lower than the mean SQI calculated from the segments of all subjects, the subject data was removed.

### Technical Validation

In order to thoroughly analyze the abundant information on cardiovascular physiology contained in the PPG signals, it was necessary to obtain the most authentic, high-precision, and high-quality PPG signal. In addition to controlling the process of data collection as accurately as possible, the PPG signal quality evaluation process was important in acquiring excellent quality signals^14,15^. This process can effectively prevent unfit waveform data from being saved and ensure that the saved data have complete heartbeat cycles, less noise, and lower drift and motion artifacts.

**Figure 4:**
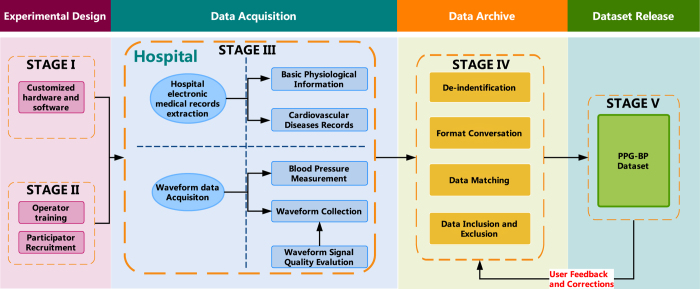
Overview of the PPG-BP Dataset.

**Figure 5:**
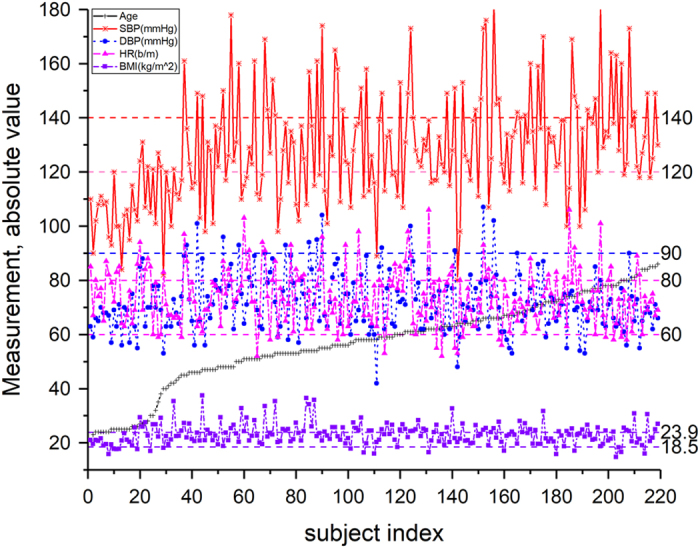
Physiological records for all subjects ordered in ascending order according to their age.

Although the potential value of the PPG signal is well known^16,17^, it is difficult to acquire authentic and rich PPG signals and to extract subtle characteristics that signify problems such as difference in body tissue^18,19^, disturbance of motion^20–23^, control of the acquisition process^24,25^, etc. This is worth more research and exploration^26^. In order to avoid or eliminate the above mentioned issues, the data collection process was conducted in a controlled, quite environment with minimal interferences. Figure 4 shows an overview of the data collection process. Figure 5 shows a statistics table of the physiological records for all subjects in the database.

The process of data collection experiment consists of five stages. The stage I and stage II conduct some preparations of the customized hardware and software, training of operators and recruitment of participants. Stage III is the phase of data collection in hospitals, including the acquisition of basic physiological information of participants, hospital electronic medical records, PPG signals and blood pressure data. Stage IV is the data archiving part, including de-identification, format conversion, data matching, data inclusion and exclusion for all the collected data. Stage V is the public release of the PPG-BP dataset; researchers can download the dataset and validate their algorithms.

At present, Perfusion Indices (P_SQI_) are regarded as the gold standard of PPG Signal Quality Indices (SQI). Various other signal quality evaluation methods have been proposed and studied in order to identify more simple and accurate evaluation methods for signal quality assessment. Elgendi^13^ compared eight different signal quality indices: P_SQI_, Skewness (S_SQI_), Kurtosis (K_SQI_), Entropy (E_SQI_), Signal-to-noise ratio (N_SQI_), Zero-crossing (Z_SQI_), Matching of multiple systolic wave detection algorithms (M_SQI_), and Relative power (R_SQI_). For varying lengths of PPG waveform recordings (i.e., from 2 s to 30 s), the S_SQI_ method demonstrated better performance when compared to other methods (P_SQI_, K_SQI_, E_SQI_, N_SQI_, M_SQI_, Z_SQI_, and R_SQI_)^13^. Moreover, PPG waveform classification is possible with 2 s length recording (excellent vs. unfit) using the S_SQI_ index^13^. These results motivated collecting PPG signals with 2 s length.

Krishnan et al.^22^ introduced and tested the Skewness statistic and found that S_SQI_ had a certain connection with the quality of PPG waveform. Other researchers validated this observation, and Elgendi ^13^ found it to be the optimal method for assessing SQI in PPG signals. Skewness is used to measure the probability distribution of symmetric signals, which can distinguish the periodic, symmetrical, stationary signals and sudden jumps, periodic signals, and irregular signals. The specific definition is as follows:

$$S_{\mathrm{SQI}}=1/N\sum_{i=1}^{N}{\left[x_{i}-{\hat{µ}}_{x}/\sigma \right]}^{3},$$

where *N* is the sample number of the PPG signal, and ${\hat{µ}}_{x}$ and *σ* are the empirical estimates of the mean and standard deviation of *x*_*i*_, respectively.

In the process of data collection, the data is evaluated using the PPG signal quality before it was saved, and the evaluation method adopted the S_SQI_ index. Each segment of PPG signal was evaluated against the classification threshold of excellent, acceptable, or unfit PPG waveform in order to determine whether it should be saved.

During the evaluation of signal quality for each participant, the S_SQI_ for the three segments were compared. Among the three the segments, the segment with the highest S_SQI_ was deemed as "high quality", the segment with the lowest S_SQI_ was deemed as "low quality" and the remaining segment was deemed as "medium quality". Note, we are providing the PPG segments and their corresponding S_SQI_ values to make it easier for investigators to select the segment with highest quality. Additionally, with the availability of the three S_SQI_ values, researchers will able to analyze each segment, if needed, for validation, etc.

### Usage Notes

The dataset is distributed in the normal standard file format (text, xlsx) and can be read and processed by a variety of packages, including Matlab, Python, and R. In addition, when using this dataset, users should first perform an effective waveform quality evaluation to select the high-quality waveform segment from the three waveform segments from each participant.

There are multiple potential uses for this dataset, the most obvious of which is to validate various CVD diseases and diabetes through pattern recognition and machine learning methods. About one-third of the dataset includes hypertensive or diabetic patients. A series of PPG characteristics can be defined and mined, the intrinsic association between characteristic and physiological process can be studied, and the prediction of blood pressure or classification of hypertension can be explored. It is important to analyze the physiological information contained in the PPG waveform comprehensively.

### Example usage

This dataset can be used to analyze the definition and extraction of morphological information of the PPG waveform. The PPG wave mainly consists of the systole period and the diastole period. At the same time, depending on different human cardiovascular health statuses, the tidal and diastolic wave may also be shown. These characteristics^27^ can be defined and extracted by means of digital signal processing such as time and frequency domain processing or wavelet transform. The time span, amplitude, area, ratio, frequency, and energy parameter are the main features that can be extracted from the PPG signal.^28^ The accurate identification and extraction of these characteristics has potential value in analyzing vascular aging, blood pressure, and arteriosclerosis, among others.

## Additional information

**How to cite this article:** Liang, Y. et al. A new, shortrecorded photoplethysmogram dataset for blood pressure monitoring in China. Sci. Data 5:180020 doi: 10.1038/sdata.2018.20 (2018).

**Publisher’s note:** Springer Nature remains neutral with regard to jurisdictional claims in published maps and institutional affiliations.

## Supplementary Material



## Figures and Tables

**Table 1:** The SQI results of all subjects’ segments

**Num.**	**subject ID**	**segment 1**	**segment 2**	**Segment 3**	**Num.**	**subject ID**	**segment 1**	**segment 2**	**Segment 3**	**Num.**	**subject ID**	**segment 1**	**segment 2**	**Segment 3**	**Num.**	**subject ID**	**segment 1**	**segment 2**	**Segment 3**
1	2	0.98	0.96	0.92	56	86	0.66	0.7	0.72	111	153	0.93	1.15	0.79	166	216	0.33	0.33	0.37
2	3	0.69	0.8	0.81	57	87	0.97	0.96	0.95	112	154	0.82	0.7	0.75	167	217	0.69	1.26	0.8
3	6	0.58	0.59	0.64	58	88	0.81	0.52	0.65	113	155	0.75	0.94	0.71	168	218	0.82	0.99	0.89
4	8	0.96	0.85	0.87	59	89	0.39	0.58	0.14	114	156	0.75	0.76	0.7	169	219	1.02	1.05	0.84
5	9	0.65	0.67	0.87	60	90	0.87	0.97	1	115	157	0.58	0.68	0.44	170	220	0.63	0.65	0.66
6	10	0.59	0.64	0.34	61	91	1.05	0.77	0.84	116	158	0.66	0.86	0.05	171	221	0.43	0.78	0.6
7	11	0.74	0.67	-0.16	62	92	0.9	1.1	1.15	117	160	0.54	0.66	0.59	172	222	0.92	0.87	0.85
8	12	0.23	0.73	0.41	63	93	0.97	0.99	0.46	118	161	0.89	0.83	0.86	173	223	0.81	0.08	0.98
9	13	0.76	0.84	1.06	64	95	1.31	0.89	0.87	119	162	1.07	1	0.97	174	224	0.85	1.03	0.75
10	14	0.77	0.72	0.15	65	96	0.75	0.89	0.81	120	163	0.79	0.42	0.61	175	226	0.84	1.04	0.44
11	15	1.23	0.77	0.3	66	97	0.56	0.42	0.76	121	164	0.94	0.85	0.71	176	227	0.99	0.88	0.94
12	16	0.64	0.66	0.83	67	98	0.88	0.98	0.86	122	165	1.07	0.96	1.02	177	228	1.06	1	0.93
13	17	0.69	0.9	0.9	68	99	0.88	0.72	0.79	123	166	0.71	0.93	0.77	178	229	1	1.09	0.98
14	18	0.87	0.59	1.05	69	100	0.58	0.66	0.16	124	167	0.57	0.76	0.57	179	230	0.59	0.72	0.81
15	19	0.78	0.19	0.16	70	103	0.37	0.4	0.44	125	169	1.11	0.82	0.91	180	231	1.28	1.46	0.98
16	21	0.65	0.74	0.75	71	104	0.88	0.23	0.85	126	170	0.77	0.85	0.95	181	232	0.92	1.21	0.87
17	22	0.73	0.44	0.39	72	105	0.54	0.97	0.8	127	171	0.69	0.46	0.48	182	233	0.89	0.86	0.67
18	23	0.7	0.6	0.74	73	106	0.82	0.9	1.16	128	172	0.45	0.56	0.53	183	234	0.62	0.81	1.02
19	24	0.74	0.74	0.75	74	107	0.89	0.58	0.66	129	173	0.89	0.84	0.97	184	235	0.94	1.08	0.97
20	25	1.38	0.15	0.78	75	108	0.71	0.69	0.64	130	174	1.14	1.03	1.17	185	237	0.79	1.19	1.42
21	26	1.31	0.47	0.83	76	110	0.9	0.83	0.88	131	175	0.69	0.62	0.71	186	239	0.81	0.8	0.7
22	27	1.86	1.33	1.26	77	111	0.9	0.85	0.76	132	176	0.75	0.73	0.68	187	240	0.6	1.09	0.93
23	29	0.73	0.6	-0.05	78	112	0.61	0.55	0.57	133	178	0.38	0.68	0.55	188	241	0.68	0.58	0.52
24	30	0.86	0.8	0.76	79	113	0.35	0.51	0.78	134	179	2.34	0.83	0.78	189	242	0.59	0.7	0.69
25	31	0.68	0.81	0.7	80	114	0.5	0.58	0.67	135	180	0.8	0.64	0.84	190	243	1.09	0.84	0.91
26	32	1.06	1.12	1.23	81	115	0.74	0.06	1.03	136	182	0.72	0.93	0.9	191	244	0.73	0.79	0.68
27	34	0.9	0.79	0.74	82	116	0.78	0.86	0.93	137	183	0.35	0.25	0.36	192	245	0.56	0.54	7.13
28	35	0.94	1.15	1.18	83	119	0.55	0.59	-0.07	138	184	0.49	0.93	0.87	193	246	1.23	1.85	0.63
29	38	0.88	0.66	0.94	84	120	0.79	0.67	0.77	139	185	1.03	1.1	1.1	194	247	0.8	0.66	0.54
30	40	0.21	1.2	0.9	85	122	0.93	0.87	0.5	140	186	0.73	0.69	0.99	195	248	0.14	0.65	0.69
31	41	0.85	0.78	0.73	86	123	0.89	0.97	1.3	141	188	0.85	1.78	0.71	196	250	0.79	0.84	0.77
32	43	0.52	0.28	0.53	87	124	0.93	1.23	1.19	142	189	0.9	0.68	0.92	197	251	0.99	0.95	0.99
33	45	0.68	0.76	0.67	88	125	0.84	-0.47	0.86	143	190	1.13	0.8	0.99	198	252	0.78	0.38	10.22
34	47	0.76	0.75	0.73	89	126	0.44	-0.01	0.54	144	191	1.23	1.1	0.85	199	253	0.8	0.89	0.92
35	48	0.74	0.79	0.63	90	127	0.53	0.83	0.75	145	192	0.85	0.87	0.8	200	254	0.51	0.84	0.75
36	50	0.79	0.72	0.73	91	128	0.87	0.86	0.9	146	193	0.76	0.53	0.63	201	256	0.95	0.72	1.25
37	51	0.6	0.2	0.49	92	130	0.91	0.97	1	147	195	0.91	1.1	0.49	202	257	0.63	0.69	0.87
38	52	0.95	0.67	0.93	93	131	0.86	0.85	0.75	148	196	0.88	0.74	0.68	203	259	0.59	0.62	0.67
39	53	0.81	0.91	0.9	94	134	0.71	0.21	0.86	149	197	0.9	1.06	1.33	204	403	0.92	0.92	0.92
40	54	1.12	1.06	1.11	95	135	0.68	0.72	0.67	150	198	1.12	1.07	1.02	205	404	1.4	1	0.96
41	55	0.63	0.99	1.1	96	136	1.73	0.56	0.8	151	199	0.81	0.96	0.83	206	405	0.72	0.79	0.96
42	56	0.26	0.4	0.47	97	137	0.28	0.58	0.79	152	200	0.3	0.86	1.02	207	406	0.28	0.45	0.54
43	57	0.69	0.59	0.65	98	138	0.74	0.48	0.59	153	201	0.68	0.69	0.8	208	407	0.84	0.82	0.76
44	58	0.97	0.62	0.75	99	139	1.35	0.69	0.63	154	203	0.92	1.05	0.86	209	409	0.84	1	0.89
45	60	0.37	1.64	0.51	100	140	0.41	0.85	0.71	155	205	0.91	0.82	0.77	210	410	0.94	0.91	0.9
46	61	0.87	0.84	0.93	101	141	1.14	0.96	0.86	156	206	0.69	0.84	0.67	211	411	1.09	0.92	1.05
47	62	0.57	0.96	0.69	102	142	0.8	0.82	0.83	157	207	0.75	0.7	0.2	212	412	0.95	0.7	0.99
48	63	1.01	0.92	0.92	103	144	0.76	0.66	-0.01	158	208	1.47	0.95	0.92	213	413	0.74	0.67	0.63
49	64	0.19	0.65	0.39	104	145	1.11	1.1	1.1	159	209	0.76	0.67	0.7	214	414	0.93	1.34	1.4
50	65	0.78	0.8	0.72	105	146	0.84	0.84	1	160	210	0.72	0.71	0.78	215	415	1.15	1.38	1.19
51	66	0.79	0.79	0.94	106	148	1.03	1.03	1.06	161	211	1.4	0.73	0.89	216	416	0.96	0.94	1.01
52	67	0.7	0.7	0.99	107	149	0.52	0.58	0.49	162	212	0.74	0.56	0.59	217	417	1.12	1.32	1.38
53	83	0.81	0.9	0.79	108	150	0.75	0.66	0.49	163	213	1.16	0.91	0.67	218	418	0.96	0.87	1.06
54	84	0.91	0.3	0.65	109	151	0.81	0.29	0.85	164	214	0.46	0.72	0.24	219	419	1.13	1	0.81
55	85	0.68	0.79	0.63	110	152	1.05	0.9	1.22	165	215	0.62	0.69	0.81					
For each subject, the highest S_SQI_ value corresponds to the signal with highest signal quality, the lowest S_SQI_ value corresponds to the signal with the lowest signal quality, and the remaining middle S_SQI_ value corresponds to the signal with the medium quality. Note, S_SQI_ stands for Skewness signal quality index.																			
